# Characterization of a Vitellogenin Receptor in the Bumblebee, *Bombus lantschouensis* (Hymenoptera, Apidae)

**DOI:** 10.3390/insects10120445

**Published:** 2019-12-12

**Authors:** Lin Du, Mingming Wang, Jilian Li, Shaoyu He, Jiaxing Huang, Jie Wu

**Affiliations:** 1 College of Animal Science and Technology, Yunnan Agricultural University, Kunming 650201, China; dulinbumblebee@sina.com (L.D.); kmhsy@163.com (S.H.); 2 Key Laboratory for Insect-Pollinator Biology of the Ministry of Agriculture and Rural Affairs, Institute of Apicultural Research, Chinese Academy of Agricultural Sciences, Beijing 100093, China; bumblebeeljl@126.com; 3 Nanchuan Bureau of Animal Husbandry and Veterinary, Chongqing 408400, China; wangmingming198561@163.com

**Keywords:** bumblebee, *Bombus lantschouensis*, vitellogenin receptor, RNA interference

## Abstract

The vitellogenin receptor (VgR) belongs to the low-density lipoprotein receptor (LDLR) family, responsible for mediating the endocytosis of vitellogenin (Vg) into the ovaries to promote ovarian growth and oviposition. Here, we cloned and measured *VgR* gene expression characteristics in the bumblebee *Bombus lantschouensis*. RNA interference was used to validate VgR function. The results showed that the full length of the *BLVgR* cDNA was 5519 bp, which included a 5280 bp open reading frame encoding 1759 amino acids (AAs). Sequence alignment revealed that the protein contained 12 LDLa, 5 EGF, 2 EGF-CA and 10 LY domains. Phylogenetic analysis showed that BLVgR and the VgR of *Bombus terrestris* clustered together and the tree of bumblebees (*Bombus*) appeared as one clade next to honeybees (*Apis*). Transcript expression analysis showed that *BLVgR* was expressed in all tested tissues and showed the highest abundance in the ovaries. *BLVgR* expression was present in all developmental stages. However, the expression level in larvae was extremely low. In addition, the expression of *BLVgR* was significantly upregulated after egg laying in both workers and queens. In new emerging workers injected with 5 µg of VgR dsRNA, the expression level of *BLVgR* was 4-fold lower than that in the GFP dsRNA-injected group after 72 h. Furthermore, *BLVgR* silencing significantly reduced the number of eggs laid (3.67 ± 1.96 eggs) and delayed the first egg-laying time (16.31 ± 2.07 days) in worker microcolonies when compared to dsGFP (37.31 ± 4.09 eggs, 8.15 ± 0.22 days) and DEPC-treated water injected controls (16.42 ± 2.24 eggs, 10.00 ± 0.37 days). In conclusion, the *BLVgR* gene and its reproductive function were explored in the bumblebee *B. lantschouensis*. This gene plays an important role in egg laying time and egg number.

## 1. Introduction

Bumblebees are one of the most efficient pollinators of many wild plants and crops, especially legumes and Solanaceae plants [1]. Moreover, they play important roles in maintaining the balance of natural ecosystems [2]. Since the 1980s, bumblebees have been used commercially to pollinate greenhouse crops around the world and have conferred significant economic benefits [3,4]. As a result, artificial bumblebee rearing has become important. Reproductive regulation is an important biological process that is vital to bumblebee artificial mass rearing. Vitellogenin (Vg) and the vitellogenin receptor (VgR) are two reproductively critical proteins that significantly affect the fecundity of bumblebees. Therefore, full elucidation of the characteristics of VgR will aid understanding of oviposition regulation.

Insect VgR belongs to the low-density lipoprotein receptor (LDLR) gene superfamily and plays a critical role in oocyte development by mediating the endocytosis of the major yolk protein precursor Vg [5]. Vitellogenin is synthesized in the fat body and is internalized by competent oocytes through membrane-bound receptors [6,7]. The main function of Vg is to provide nutriment for embryos. Moreover, it can protect workers and the queen from oxidative stress that contributes to longevity [8,9]. Interestingly, recent studies have shown that Vg expression also affects the behavior of social insects, as it is related to division of labor [9,10]. Insect VgRs and all other LDLR family receptors share five structural domains: the ligand-binding domain (LBD), made up of cysteine-rich repeats; the epidermal growth factor (EGF)-precursor homology domain, characterized by a recurrent ‘YWXD’ motif (YWXD repeats); the O-linked sugar domain of unknown function; the transmembrane domain anchoring the receptor in the plasma membrane; and the cytoplasmic domain containing an internalization signal [5]. In 1986, the VgR protein from the oocytes of the locust *Locusta migratoria* was first identified and purified [11]. Since then, it has been identified in a variety of economic insects [12,13] and agricultural pests [14,15,16,17]. In 2004, the cloning of the first hymenopteran *VgR* cDNA from the imported fire ant *Solenopsis invicta* (SiVgR) was reported, and this *VgR* was upregulated by methoprene, a juvenile hormone (JH) analog [18]. In 2006, RNAi technology was first used to analyze VgR function in the cockroach *Blattella germanica* [19]. However, the first silencing of hymenoptera VgR with RNAi was achieved in 2009 [20]. The expression characteristics of VgR from the honeybee *Apis mellifera* in the queen and workers were also studied, and the results suggested that VgR is not caste or tissue specific. It was detected in all sexes and outside the ovary [21]. To date, the characteristics of VgR have not been explored in bumblebees.

RNA interference is a technology for posttranscriptional gene silencing that has been developed as a powerful tool for studying gene function in a variety of organisms [22]. A variety of efficient means to transport double-stranded RNA (dsRNA) into organisms has been developed including microinjection, feeding and transgenic plant expression [23,24,25]. RNAi has achieved prodigious progress in insect pest control and economic insect biology research [20,25]. In recent years, RNAi has been used to study the regulation of target gene expression, caste differentiation and disease control in honeybees [26,27,28]. However, there have been no reports on reproductive inhibition by using RNAi in bumblebees.

The bumblebee *Bombus lantschouensis* is a polylectic and primitively eusocial bee that is widely distributed in medium-elevation mountains and plateaus in northern China [29]. It is an important pollinator of wild plants and crops and has been selected as the native species applied to pollinate greenhouse crops. A recent study indicates that the pollination efficiency of this species exceeds that of honeybees. Interestingly, *B. lantschouensis* prefers to visit plants with less pollen and nectar than honeybees [30]. The molecular mechanisms of reproduction, such as the female fertility regulation, are a vital aspect of artificial mass rearing. Vitellogenin and the vitellogenin receptor are important genes for insect fecundity [6]. Therefore, it is necessary to explore the effect of BLVgR on female reproduction in *B. lantschouensis*.

Understanding the molecular mechanisms of reproductive regulation is critical for bumblebee artificial rearing. In the present study, the Vg transporter protein VgR gene of a bumblebee (*B. lantschouensis*) was cloned, and its expression profile was checked in different tissues, developmental stages and reproductive statuses. RNAi was used to validate the function of *BLVgR* in ovarian activation, first egg laying time and number of eggs laid. These results help us to understand the basic knowledge of VgR in bumblebee commercial rearing.

## 2. Materials and Methods

### 2.1. Samples

Bumblebees (*B. lantschouensis*) were collected from the rearing room at the Institute of Apicultural Research, Chinese Academy of Agricultural Science, Beijing, China. Bumblebees were sampled from thirty independent colonies that were raised in an artificial breeding room (in constant darkness with a temperature of 28 ± 0.5 °C and 60 ± 5% relative humidity) and fed fresh frozen pollen and a 50% sugar solution every other day [31]. Different tissues from 18 egg-laying queens were sampled. The tissues were as follows: antenna (AN), head (HD), thorax (TH), leg (LG), epidermis (EP), midgut (MG), ovary (OV), fat body (FB) and venom gland (VG). All tissues were dissected and frozen in liquid nitrogen immediately. Tissues from six bees as a biological replicate were pooled, and total RNA was extracted. Samples from three different development stages except the egg stage of bumblebee workers, were obtained individually at the following stages: within 48 h of larval hatching (Lar), pupae with an unpigmented body cuticle and white eyes (Pw), pink eyes (Pp), and brown eyes (Pb), pupae with dark brown eyes and a light-pigmented thorax (Pbl), and pupae with brown eyes and a dark-pigmented cuticle (Pbd). Eggs were sampled within 24 h, eighteen eggs were collected (six eggs in each biological replicate). The expression level of VgR in the ovary was measured at egg-laying and non-egg-laying statuses of queens and workers. Each reproductive status had three samples [32]. All samples were stored at −80 °C until use.

### 2.2. RNA Extraction and Synthesis of cDNA

Total RNA was extracted from all samples with TRIzol reagent (Invitrogen, Carlsbad, CA, USA) following the manufacturer’s instructions. The purity of the RNA was assessed using a NanoDrop 2000 spectrophotometer (Thermo Fisher Scientific, Waltham, MA, USA) at 260/280 nm, and RNA integrity was screened by 1.5% (w/v) agarose gel electrophoresis. The first strand of cDNA was synthesized according to the instructions of the Reverse Transcription kit (Takara, Dalian, China). The reaction conditions were as follows: 42 °C for 30 min, 99 °C for 5 min, and 5 °C for 5 min, after which the product was stored at −20 °C until use.

### 2.3. Molecular Cloning of BLVgR

To obtain a specific fragment of the *BLVgR* cDNA, primers (Table 1) were designed and synthesized according to the predicted *VgR* sequence of *Bombus terrestris* (GenBank accession number: LOC100649042). The PCR amplification conditions were as follows: 94 °C for 3 min, 35 cycles of 94 °C for 30 s, 55 °C for 50 s and 72 °C for 1 min, and 72 °C for 10 min. The PCR products were ligated into the pMD19-T vector (Takara Bio Inc., Dalian, China), which was then transformed into Trans1-T1 *E. coli* competent cells (Transgen Biotech, Beijing, China). Ampicillin resistance and white-blue screening were used to select the positive clone. Positive colonies were sent for sequencing by the Sino GenoMax Company (Beijing, China) using the Sanger method.

Full-length cDNA amplification was performed using a SMARTer rapid amplification of cDNA ends (RACE) amplification kit (Clontech, Mountain View, CA, USA) according to the manufacturer’s recommendations with the appropriate primers (Table 1). PCR was carried out with 5 cycles of 94 °C for 30 s and 72 °C for 3 min; 5 cycles of 94 °C for 30 s, 70 °C for 30 s and 72 °C for 3 min; and 27 cycles of 94 °C for 30 s, 68 °C for 30 s and 72 °C for 3 min. The amplified DNA fragment was cloned and sequenced to both ends.

### 2.4. Sequence Analysis

Sequences were assembled, and the open reading frame (ORF) was predicted using BioEdit 7.2.5 software (http://www.mbio.ncsu.edu/BioEdit/bioedit.html). The molecular weight (MW), theoretical isoelectric point (pI), properties of the amino acid residues, and protein hydropathy were assessed using the European Molecular Biology Open Software Suite (EMBOSS) (http://emboss.sourceforge.net/) [33]. The online TMHMM-2.0 program was used to analyze the protein transmembrane domain (http://www.cbs.dtu.dk/services/TMHMM-2.0/) [34]. The signal peptide position was predicted using SignalP-5.0 (http://www.cbs.dtu.dk/services/SignalP-5.0/) [35]. We download homologous VgR amino acid sequences from GenBank, including those from the following species: *B. terrestris* (XP_003402703.1), *Bombus impatiens* (XP_012241122.1), *Apis cerana* (PBC31775.1), *Apis mellifera* (XP_026295652.1), *Apis dorsata* (XP_006610571.1), *Apis florea* (XP_012350206.1), *Solenopsis invicta* (NP_001291525.1), *Nasonia vitripennis* (XP_008217630.1), *Drosophila melanogaster* (NP_996433.1), *Drosophila sechellia* (XP_002042769.1), *Drosophila yakuba* (XP_002100545.2), *Drosophila erecta* (XP_001978195.1), *Drosophila ananassae* (XP_001966853.1), *Drosophila persimilis* (EDW29780.1), *Drosophila pseudoobscura* (XP_001354886.2), *Drosophila willistoni* (XP_002064208.2), *Drosophila virilis* (XP_002054982.2), *Aedes aegypti* (AAC28497.1), *Anopheles gambiae* (XP_310672.5), *Blattella germanica* (CAJ19121.1), *Rhyparobia maderae* (BAE93218.1), *Periplaneta americana* (BAC02725.2), and *Acyrthosiphon pisum* (XP_016657813.1). The SMART program (http://smart.embl-heidelberg.de/) was used to identify conserved domains [36]. Evolutionary analyses were conducted in MEGA X [37]. The VgR sequence of *A. pisum* was used as an outgroup. The evolutionary history was inferred by using the maximum likelihood method and general reversible chloroplast plus frequency model, and the reliability of the tree was assessed with 1000 bootstrap replications. Branches corresponding to partitions reproduced in less than 50% bootstrap replicates are collapsed [38].

### 2.5. BLVgR Expression Analysis Using Quantitative RT-PCR

The expression levels of *BLVgR* mRNA in different tissues, developmental stages and reproductive status of workers and queens, as well as the results of different RNAi treatments, were analyzed by real-time quantitative PCR using a Stratagene Mx3000 real-time PCR system (Agilent, USA). First-strand cDNA samples, were diluted (1:10 *v*/*v*) with DEPC-treated water. Amplification was carried out in a 20 µL reaction volume containing 10 µL of 10 × TB Green Master Mix (Takara, Dalian, China), 2 µL cDNA, and 0.5 µL of each 10 µm primer (Table 1). Quantitative measurements were normalized using *β*-actin and GAPDH. The qPCR conditions were as follows 95 °C for 30 s, followed by 40 cycles of 95 °C for 5 s, and 63 °C for 1 min. Each assay was performed in triplicate and repeated with three independent samples. The relative quantities of *BLVgR* transcripts were calculated using the comparative Ct method [39].

### 2.6. RNA Interference

To synthesize double-stranded RNA (dsRNA), a specific fragment was amplified with two T7 promoter primers (Table 1). The amplification conditions were predenaturing at 94 °C for 5 min, followed by 35 cycles of denaturing at 94 °C for 30 s, annealing at 58 °C for 1 min and extension at 72 °C for 1 min, with a final extension at 72 °C for 10 min. The GFP gene (ACY56286) and DEPC-treated water were used as a negative control [17,23,40]. Double stranded RNA was synthesized using the T7 RiboMAX™ Express RNAi System (Promega, Madison, WI, USA) according to the manufacturer’s suggestions. The dsRNA was dissolved in the appropriate volume of DEPC-treated water and stored at −80 °C.

Newly emerged workers (within 24 h) from five colonies were injected with 10 µL (500 ng/µL) of a dsRNA solution containing dsVgR (N = 93), dsGFP (N = 93) or DEPC-treated water (N = 114). The bees were injected through the conjunction between the 5th and 6th abdominal sternum using a FemtoJet micro-injector [41]. After injection, three bees were selected randomly to form a micro-colony individually (21 × 14 × 7 cm contained four colonies to share the food). To obtain the optimal dose of microinjection, eighteen microcolonies including nine colonies of dsVgR and nine colonies of dsGFP were used to test three different concentrations (0.2, 1 and 5 µg) of dsRNA with three biological replicates. The expression of VgR in the ovary was used to measure the silencing efficiency with qPCR at 72 h after the injection. The concentration of 5 µg was the best silencing efficiency and this concentration was used to evaluate the effect of silencing on egg laying. The number of eggs laid within 15 days and the first egg laying time were counted and recorded based on 13 microcolonies of dsVgR (39 workers), 13 microcolonies of dsGFP (39 workers) and 19 microcolonies of DEPC-treated water (57 workers) after injection. The laying status was checked every day. When the worker laid eggs, we removed the eggs and recorded the number of eggs. The microcolonies that remained were used to observe the development of the ovaries.

### 2.7. Data Analysis

All data are presented as mean ± SE. Before the statistical test, the data were tested for normality. The difference of treatments was analyzed by one-way analysis of variance (ANOVA) followed by Tukey post hoc test with R-project software version 3.5.1 (https://cran.r-project.org).

## 3. Results

### 3.1. Full-Length cDNA of BLVgR

**Figure 1:**
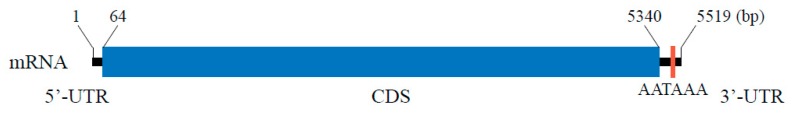
mRNA structure of *BLVgR* in the bumblebee (*Bombus lantschouensis*). The numbers indicate the position in the sequence. The full-length BLVgR cDNA sequence is 5519 bp. The 5′ UTR and 3′ UTR are 63 and 179 bp. A polyadenylation signal (AATAAA) is found at the end of the mRNA sequence.

The specific amplification fragments and the 3′ end and 5′ end RACE sequences were merged with BioEdit. The results showed that the full-length cDNA of *BLVgR* (GenBank ID: MN217253) was 5519 bp and contained a 63 bp 5′ UTR and 179 bp 3′ UTR with a polyadenylation signal (AATAAA) (Figure 1). The ORF was composed of 5280 nucleotides encoding 1759 amino acids. The initiation codon (ATG) and stop codon (TAG) were present at positions 64–66 and 5341–5343, respectively. The predicted molecular weight of *BLVgR* was 198.131 kDa, and the theoretical pI was 5.9. Moreover, *BLVgR* contained a signal peptide with a potential cleavage site between amino acid residues 27 and 28.

### 3.2. Sequence and Phylogenetic Analysis of BLVgR

**Figure 2:**
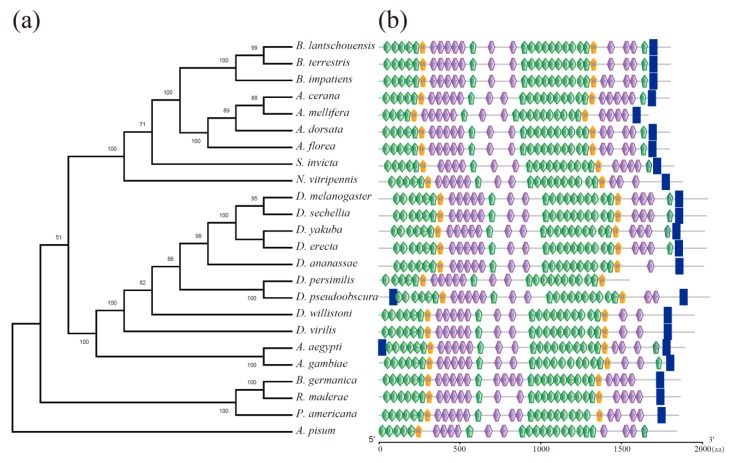
Diagrammatic phylogenetic relationship and structure comparisons of BLVgR with sequences from other insects: (**a**) Phylogenetic analysis of BLVgR with the vitellogenin receptors of insects from different orders. The phylogenetic tree was constructed by using MEGA X with a statistical method of maximum-likelihood. The VgR sequence of *A. pisum* was used as an outgroup. The numbers indicate bootstrap support values (%) based on 1000 replicates. Branches corresponding to partitions reproduced in less than 50% bootstrap replicates are collapsed. (**b**) Comparison of BLVgR modular domains with those of other insects. LDLa, low-density lipoprotein receptor domain class A (hexagon with green); EGF, epidermal growth factor-like domain (pentagon with green); EGF_CA, calcium-binding EGF-like domain (pentagon with yellow); LY, low-density lipoprotein receptor YWTD protein (hexagon with purple); transmembrane domain (tetragon with blue).

The analysis of conserved domains showed that BLVgR is a member of the LDLR superfamily of receptors, and the BLVgR protein was most similar to the VgR protein sequence of *B. terrestris* (XP_003402703.1), followed by that of *B. impatiens* (XP_012241122.1), as expected due to their similar taxonomic positions (Figure 2). BLVgR exhibited two ligand-binding domains (LBDs) with four class A cysteine-rich repeats (LDLa) in the first domain (LBD1) and eight repeats in the second domain (LBD2). Each LBD was followed by an epidermal growth factor (EGF)-like domain. The first EGF-like domain contained a calcium-binding domain, seven LY domains and three EGF domains, whereas only a calcium-binding domain, three LY domains and two EGF domains were found in the second. The second EGF-like domain was followed by a transmembrane domain and a cytoplasmic domain (Figure 2). Therefore, this sequence is considered to be the full-length cDNA sequence of *B. lantschouensis*.

To inspect the evolutionary relationships between BLVgR and other VgRs from different species, a phylogenetic tree of VgRs was constructed (Figure 2). As expected, VgRs of bumblebees (*Bombus*) appeared as one clade next to honeybees (*Apis*), and an ant (*Solenopsis*) was closer to the bees than the parasitic wasp (*Nasonia*).

### 3.3. Expression Patterns of BLVgRs in Different Tissues, Developmental Stages and Reproductive Status

**Figure 3:**
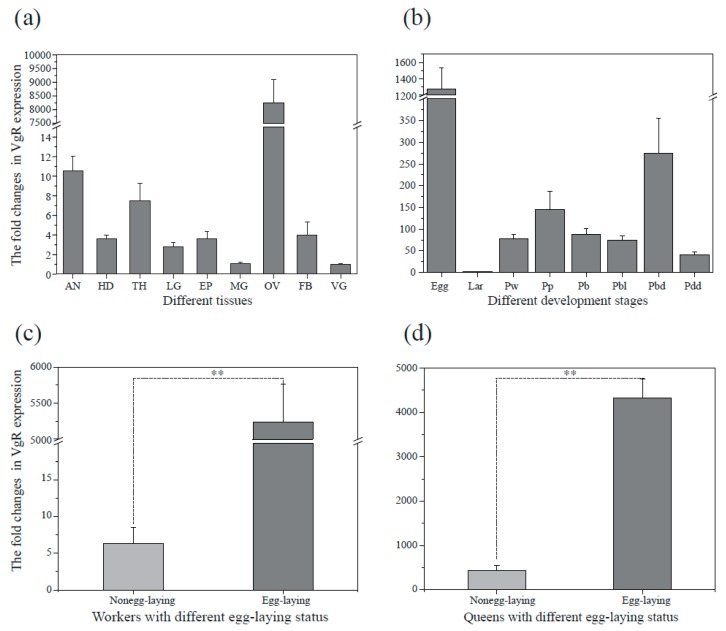
Analysis of relative *BLVgR* expression in different tissues, developmental stages and reproductive status in bumblebees (*B. lantschouensis*). (**a**) Analysis of relative *BLVgR* expression in different tissues: antenna (AN), head (HD), thorax (TH), leg (LG), epidermis (EP), midgut (MG), ovary (OV), fat body (FB), and venom gland (VG) (F = 91.811, df = (8, 72), *p* < 0.05). (**b**) Analysis of relative *BLVgR* expression at different developmental stages: Egg (within 24 h of laying), Lar (within 48 h of larvae after hatching), Pw (white-eyed pupae with an unpigmented cuticle), Pp (pink-eyed pupae with an unpigmented cuticle), Pb (brown-eyed pupae with an unpigmented cuticle), Pbl (brown-eyed pupae with thoracic pigmentation), Pbd (brown eyes with a dark-pigmented cuticle), and Pdd (dark-eye pupae with a dark-pigmented cuticle) (F = 18.373, df = (7, 64), *p* < 0.05). (**c**) Differences in the expression of the *BLVgR* gene in *B. lantschouensis* workers with different reproductive status. Three egg-laying and non-egg-laying workers were sampled. The expression level of *BLVgR* was significantly increased in egg-laying workers compared with non-egg-laying workers (F = 95.588, df = (1, 16), *p* < 0.01). (**d**) Differences in the expression of the *BLVgR* gene in queens with different reproductive status. Three egg-laying and non-egg-laying queens were sampled. The expression level of *BLVgR* was significantly increased in egg-laying queens compared with non-egg-laying queens (F = 48.977, df = (1, 16), *p* < 0.01). The asterisks above the error bar indicate that the expression levels were significantly different (*p* < 0.01).

The BlVgR transcript was detected in all tissues, and the highest transcript levels were detected in the ovary. Except in the ovary, the expression level was not significantly different between different tissues (*p* > 0.05) (Figure 3a). Between the various developmental stages, relatively higher transcript abundance of *BLVgR* was observed in the eggs than in the other developmental stages. However, it dropped to the lowest level at the larval stage. There were no significant differences between any pupal stages (Figure 3b). For both castes, queens and workers, individuals who were laying eggs had significantly higher transcript levels of *BLVgR* than non-laying individuals (*p* < 0.01; Figure 3c,d). Therefore, *BLVgR* was positively correlated with an enhanced female reproductive status.

### 3.4. RNA Interference

**Figure 4:**
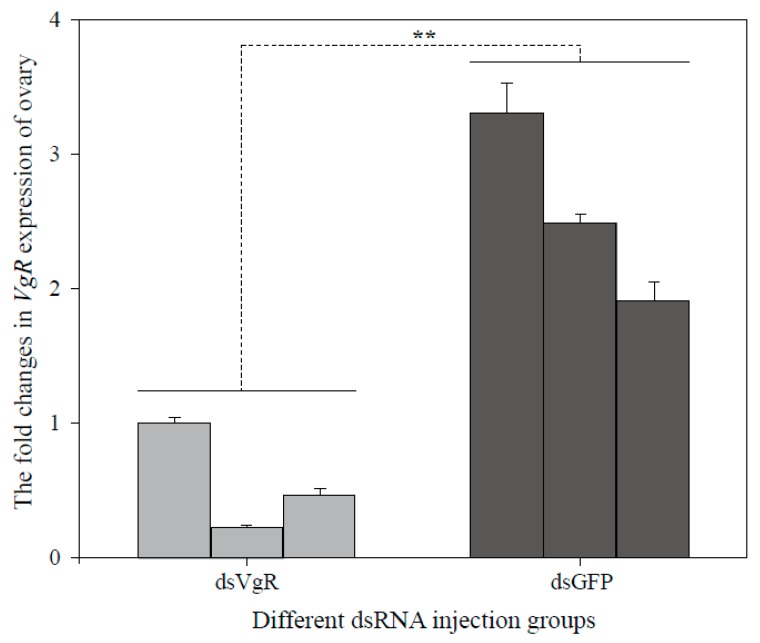
The transcript level of *VgR* in ovaries of bumblebee workers that were subjected to RNA interference. The expression levels of *BLVgR* were determined by qPCR following the injection of dsVgR after 72 h. Three microcolonies of each treatment (dsVgR and dsGFP) were sampled. The dsGFP treatment group was used as a control. The expression level of VgR was significantly decreased (** *p* < 0.01).

**Figure 5:**
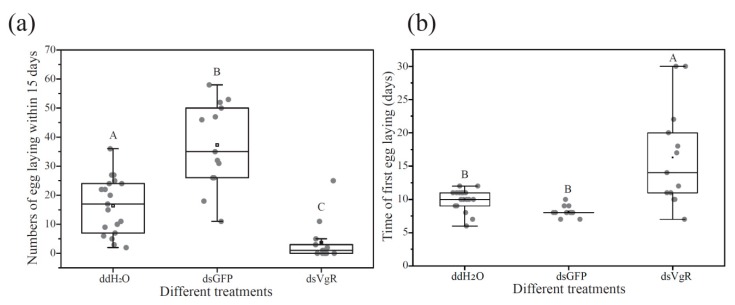
The fecundity including the number of eggs laid and the first egg-laying time was evaluated after dsRNA RNAi-mediated knockdown of VgR. The total number of eggs laid in each microcolony was counted within 15 days after injection. The number of microcolonies in dsVgR, dsGFP and DEPC-treated water groups was 13, 13, and 19. (**a**) The difference of egg laying number under different treatments. Eggs were removed every day and the number was counted. (**b**) The difference of first egg laying time under different treatments. Egg laying status was checked every day. When the first egg was observed, the first egg laying time was recorded. Different letters represent a significant level (*p* < 0.01).

**Figure 6:**
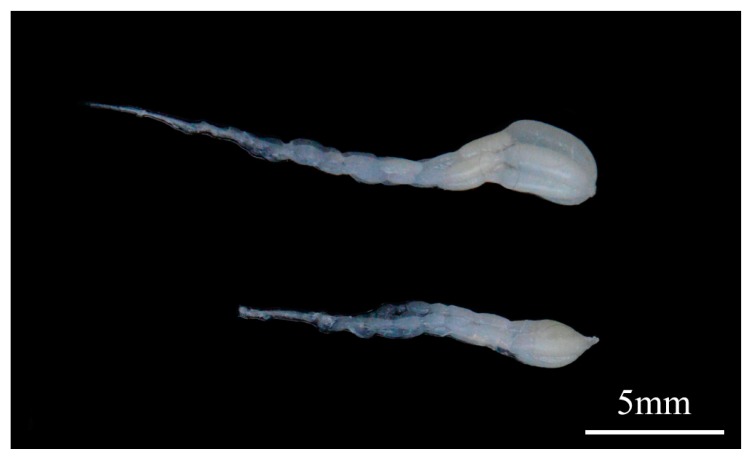
Ovary development status was evaluated after the RNAi-mediated knockdown of *BLVgR*. Newly emerged adult workers were injected with dsGFP and dsVgR. Ovaries were dissected under dissection microscopy and photographed with a NIKON (D750) camera 7 days after the injection. The ovary on the top was from the dsGFP group, and the ovary on the bottom was from the dsVgR group.

To explore the efficiency of dsRNA injection, *BLVgR* expression levels were measured by qPCR. The *BLVgR* transcript levels of dsVgR-injected workers were significantly decreased by 4-fold compared to those of the dsGFP group at 72 h after dsRNA injection (Figure 4). The number of eggs laid by workers within 15 days after injection was significantly decreased in the dsVgR group compared to the dsGFP (F = 54.685, df = (1, 24), *p* < 0.01) and DEPC-treated water (F = 16.1448, df = (1, 24), *p* < 0.01). The numbers of eggs were lower by 74.5% and 90.1% in the workers injected with dsVgR compared to the DEPC-treated water and dsGFP groups, respectively (Figure 5a). Moreover, the time until first egg laying in the dsVgR-treated workers was significantly longer than in the dsGFP (F = 15.287, df = (1, 24), *p* < 0.01) and DEPC-treated groups (F = 12.854, df = (1, 30), *p* < 0.01) (16.31 ± 2.07, 8.15 ± 0.22, and 10.00 ± 0.37 days, respectively). Colonies treated with dsVgR were delayed 8 d and 6 d compared to the colonies treated with dsGFP and DEPC-treated water (Figure 5b). The dissection of ovaries showed the degree of ovary swelling was lower compared to that in the dsGFP groups (Figure 6).

## 4. Discussion

The understanding of the molecular mechanisms of insect reproductive regulation has reached a new level, especially in social insects [20]. The bumblebee, *B. lantschouensis* is a social bee that is endemic to northern China and has been selected for artificial rearing and crop pollination [29,32,42]. The development of bee colonies is highly dependent on the ovary activation in the queen, in which the biosynthesis and transport of Vg are vital [43]. VgR as a transporter of Vg, is essential for ovary activation. Moreover, VgR has been demonstrated to not only mediate the transport of Vg but also play an important role in vitellogenesis [18,44]. In the present study, the complete *VgR* cDNA sequence of *B. lantschouensis* was identified, which, to our knowledge, represents the first report of a VgR from a bumblebee. It was demonstated that BLVgR affects the first egg-laying duration and the number of eggs laid in workers. These findings will benefit from studying the mechanism of vitellogenin transport and absorption in bumblebees.

The domains present in BLVgR indicate that BLVgR is a member of the LDLR family (Figure 2). BLVgR was shown to contain two ligand-binding domains with four and eight LDLa repeats, respectively, and the BLVgR LDLa repeats present an architecture that is closer to the VgR architecture of *A. mellifera*, *S. invicta* and *N. vitripennis* [18,21]. However, the number of LDLa repeats in Hymenoptera differ from that in other insects. For example, there are four and seven LDLa repeats in Lepidoptera [12,15], five and eight repeats in Diptera and Blattaria [45]. Additionally, BLVgR contains two EGF-like domains, both of which include a calcium-binding site similar to the VgRs of *A. mellifera*, *S. invicta* and *D. melanogaster*. The O-linked sugar domain is in the last EGF-like domain and the TM domain [5,46]. In this study, no O-linked sugar domain was found in BLVgR, which is consistent with the VgRs of *B. dorsalis* and *S. invicta* [18,47], and is different from *A. mellifera*, *L. maderae*, and *N. lugens* [17,21,45]. This indicates that the O-linked sugar domain motif is not the common functional domain of vertebrate VgRs [48].

For long time, it was assumed that vitellogenin receptor expression is highly tissue specific and is restricted to the ovaries of the females of insects [15,17,45]. However, it was found that the *AmVgR* of *A. mellifera* is expressed not only in the ovarian tissues of reproductive females but also in males [21]. This situation contrasts with observations made in *S. invicta* in which VgR expression is limited to the ovaries [20]. In the present study, *BLVgR* was expressed in all tested tissues and showed the highest abundance in the ovaries, which may be related to the pleiotropic biological functions of its ligand Vg (Figure 3c). Besides the reproductive process, it has been demonstrated that vitellogenin is also involved in regulating the longevity of the queen and workers through epidemic inhibition of the antioxidant response [49]. In addition, it is also related to the regulation of JH titer and co-opted to regulate division of labor in workers [8]. However, recent studies have found that the LDLR family receptor has the function of signal transmission, which directly transmits signals on the target cell’s plasma membrane by interacting with the cytoplasmic adaptor and the scaffold protein [50,51]. Therefore, the tissue-specific expression of BLVgR may be related to the alternative function of its ligand and the multi-function of the receptor.

The expression of *BLVgR* gene was irregular throughout stages before emerging. The high expression at the egg, PW and Pbd stage suggest that it is essential to metamorphosis in these stages. In addition, its almost disappearance in the larval stage means the function of VgR is minor. Interestingly, our results are consistent with the Vg expression changes of the bumblebee, *Bombus hypocrita* [52]. The expression of Vg mRNA in females was detected at the Pw stage, and increased during the entire pupal development with a peak at the Pbd stage. This suggests that the transcriptional changes of VgR is related to it ligand concentration.

*BLVgR* expression was closely correlated with the ovarian development of adult females. Significant differences in the expression of *BLVgR* were observed in different egg-laying statuses. The levels of *BLVgR* transcripts were increased in the ovaries of egg-laying queens and workers in comparison to virgin queens and naïve workers (Figure 3c,d). In *A. mellifera*, the *AmVgR* transcript level was increased in the ovaries of egg-laying queens in comparison to virgin queens as well as workers [21]. In *A. aegypti*, VgR transcript levels in the ovaries rapidly increase after adult eclosion and continue to rise as the ovaries become vitelogenic, reaching peak levels 24 h after a blood meal [53]. However, in cockroaches, the developmental profile of VgR shows the highest levels in the immature ovaries of final-instar female nymphs and in the early previtellogenic period [45]. Moreover, the *SiVgR* transcript is present at higher levels in virgin alate females than in reproductively active queens in *S. invicta* [18]. The different spatiotemporal dynamics of VgR may be caused by the different functions of VgR in different organisms such as observed in A. mellifera [21].

RNAi is a powerful tool for exploring gene function [16]. In 2009, the first study achieving successful posttranscriptional silencing of *VgR* was reported in Hymenoptera [20]. In bumblebees, RNAi is an effective method for gene function verification. Kim et al. demonstrated expression regulation by reducing Bi-Tf or Bi-FerHCH levels in the fat body via RNA interference in bumblebee [54]. Silencing of *VgR* in *S. invicta* led to a significant decrease in *SiVgR* expression in queen ovaries derived from dsVgR-injected pupae and stunted oocytes showing no *VgR* signal; the blocking effect continued for at least 10 days upon the eclosion of virgin queens [20]. Interestingly, the silencing of *VgR* in cockroaches significantly decreased *NlVgR* transcript and protein levels after injection with dsRNA, and inhibited Vg endocytosis in the oocytes. Moreover, silencing *NlVgR* causes a delay in ovarian development and inhibition of oviposition [17]. In this study, newly emerged workers and 5 µg dsVgR per bee were selected as the appropriate stage and the optimal dose for microinjection. After dsVgR treatment, the mRNA expression of *BLVgR* was significantly lower than in the dsGFP group (Figure 4). As expected, the number of eggs laid in the dsVgR-treated groups was lower than that in the controls (Figure 5a). In contrast, the time to the first egg laying was longer (Figure 5b). Similar results were observed in *H. armigera* [15] and *N. lugens* [17]. However, the difference in egg production between the dsGFP group and the DEPC group was significant in our study. This may be caused by the injection of dsGFP that lead to the high expression of immune genes and the high expression of immune genes will benefit egg laying [55,56].

In summary, we have demonstrated that RNAi can successfully silence *BLVgR* to affect the fertility of female bumblebees. However, this study examined the function of VgR in worker bees without examining queens. Both workers and queens develop from diploid fertilized eggs and share the same genes. It has been reported in *B. terrestris* that the microcolonies (groups of three workers isolated from the queen) can be used to estimate egg numbers, the delay before egg laying, larval production, and adult offspring fitness [57]. Microcolonies are also used to measure the lethal and sublethal effects of insecticidal proteins expressed in transgenic plants, as well as growth efficiency associated with different pollen types in bumblebees [58,59]. In conclusion, regardless of essential characteristics, this study clearly indicates that VgR plays an important role in female oviposition and ovarian maturation in bumblebees.

## 5. Conclusions

In conclusion, we identified the cDNA and analyzed the molecular characteristics of *BLVgR* from the bumblebee *B. lantschouensis*. This gene is structurally conserved compared with the homologous genes of other species. The expression patterns of *BLVgR* in different tissues, development stages, and reproductive status were identified. In addition, the function of *BLVgR* was verified by RNAi technology. The injection of dsVgR significantly reduced VgR expression, and worker ovarian development was affected, oviposition was delayed, and egg production was remarkably decreased. Our study provides insights into the reproductive regulation of fecundity in bumblebees. Therefore, the results of the present study provide basic knowledge of the reproductive biology of bumblebees.

## Figures and Tables

**Table 1:** Primers used for the identification and analysis of the *B. lantschouensis VgR* (*BLVgR*).

Purpose	Name	Primer Sequence (5′–3′)
cDNA cloning	VgRF	ACTCATGTTTGTGCCAACCTG
	VgRR	TTGGATGGTACAGATCGAAGG
RACE	5′VgR9F	TCGAGGTGCAAGCATGAGGATCAGTT
	3′VgR9R	GCGTTTCTTTGGCATGTTACGCTCT
	UPM-Long	CTAATACGACTCACTATAGC
	UPM-Short	TCACCGCATTCATCTTCC
Real-time PCR	FVgRF	GTGTGCCTGTTATCTAATGCTGAT
	FVgRR	TTCATCTTCACCGTTAGGACAATC
	β-actinF	CGACTACCTCATGAAGATT
	β-actinR	CGACGTAACAAAGTTTCTC
	GAPDH-F	GCTGGAGCTGAATATGTTGTAGAATC
	GAPDH-R	AGTAGTGCAGGAAGCATTAGAGATAACT
RNAi	RNAiVgR1F	GTTTCAATGTAAAAACGGCGACT
	RNAiVgR1R	TCGTTCTTTGGACAATCTGTAACG
	T7RNAiVgR1F	GGATCCTAATACGACTCACTATAGGGTTTCAATGTAAAAACGGCGACT
	T7RNAiVgR1R	GGATCCTAATACGACTCACTATAGGTCGTTCTTTGGACAATCTGTAACG
	GFP-F	CCACAAGTTCAGCGTGTCCG
	GFP-R	AAGTTCACCTTGATGCCGTTCT
	T7GFP-F	GGATCCTAATACGACTCACTATAGCCACAAGTTCAGCGTGTCCG
	T7GFP-R	GGATCCTAATACGACTCACTATAGAAGTTCACCTTGATGCCGTTCT
